# Hearing aid verification: Practices and perceptions of South African audiologists

**DOI:** 10.4102/sajcd.v71i1.1059

**Published:** 2024-12-09

**Authors:** Jared Moll, Zani Burger, Daneel M.P. Jacobs, Retshepisitswe P. Mothibe, De Wet Swanepoel, Faheema Mahomed-Asmail

**Affiliations:** 1 Department of Speech-Language Pathology and Audiology, Faculty of Humanities, University of Pretoria, Pretoria, South Africa; 2 Department of Otolaryngology – Head and Neck Surgery, University of Colorado School of Medicine, Aurora, United States of America; 3 Virtual Hearing Lab, a Collaborative initiative between the University of Colorado School of Medicine, Aurora, Colorado, United States of America

**Keywords:** hearing aids, verification, practices, perceptions, real ear measurements, resources, standards

## Abstract

**Background:**

Hearing aid verification is required to objectively measure hearing aid outputs by ensuring that the amplified speech spectrum closely approximates the prescription goals.

**Objectives:**

This study aimed to determine audiologists’ perceptions and practices regarding hearing aid verification and identify facilitators and barriers to its use.

**Method:**

A cross-sectional national e-survey included questions related to demographics, perceptions of verification, verification practices and two open-ended questions regarding the facilitators and barriers to conducting hearing aid verification in South Africa.

**Results:**

Seventy-eight South African audiologists, with experience ranging from less than a year to 34 years, completed the online survey. Of these, 76.3% conduct hearing aid verification, while 23.7% seldom or never perform this verification. Among the audiologists who conducted verification, 81.0% reported performing it on both adults and children. More than three-quarters (86.6%) indicated that they conduct verification only during the initial fitting, while half (53.8%) do so only when a problem arises. Thematic analysis revealed the following barriers to verification: improper equipment, a lack of equipment, non-standard clinical practices and time constraints.

**Conclusion:**

There are several challenges faced by South African audiologists in performing verification. It may be feasible to address these barriers by raising awareness about the value of verification, offering training, and advocating for the purchase and utilisation of verification equipment.

**Contribution:**

This study’s findings provide information on the current practices of hearing aid verification in a socioeconomically diverse setting. Furthermore, it highlights important challenges such as a lack of equipment as well as time constraints.

## Introduction

Hearing loss is a pervasive global health concern affecting approximately 432 million adults, constituting more than 5% of the world’s population (World Health Organization, 2023). For individuals struggling with disabling hearing loss (HL), hearing aids (HA) serve as a primary clinical intervention (Ferguson et al., 2017). By amplifying and enhancing access to sound, especially speech sounds, hearing aids mitigate the adverse consequences of hearing loss and foster greater participation in daily activities (Ferguson et al., 2017; Jorgensen, 2016). Recognised as a cornerstone of hearing rehabilitation, hearing aid fitting involves adherence to guidelines that recommend in-person verification procedures (Mueller et al., 2017). Verification, also known as real-ear measurements (REMs), has been a vital process through which audiologists determine the extent to which hearing aids meet specific client expectations and output goals, which involves objective and accurate measurements of hearing aid output (Jorgensen, 2016).

The verification process serves as a quality control measure and is widely endorsed by professional societies, including the British Society of Audiology, the American Speech-Language-Hearing Association (ASHA), the American Academy of Audiology (AAA) and others (Almufarrij et al., 2021). Audiology best practice guidelines emphasise completing REMs as part of routine patient care (Jorgensen, 2016).

To verify a hearing aid fitting, the audiologist first needs to confirm which hearing aids (HA) parameters apply to the specific individual (e.g. hearing aid type, tube size, moulds, venting, etc.) and select the appropriate fitting formula (DSL v5 and NAL-NL2) (Mueller & Picou, 2010). This selection process is necessary to ensure that appropriate gain is applied to the incoming acoustic signal, thereby ensuring the best outcome for the hearing aid user (Mahomed-Asmail, Le Roux, Laurent, 2016). Conducting REMs offers numerous advantages including improved speech recognition, enhanced speech intelligibility, increased speech perception in noisy environments and enhanced patient satisfaction (Almufarrij et al., 2021; Jorgensen, 2016; Kochkin et al., 2010). Two studies have indicated that the initial fit estimated by the hearing aid software notably deviates from prescriptive targets resulting from inadequate match to gain and frequency response slope, which can be addressed using REMs (Almufarrij et al., 2021; Munro et al., 2015).

Despite these deviations, a review by Almufarrij et al. (2021) identified three separate studies where a significantly higher number of participants preferred their hearing aids after REMs were performed (Almufarrij et al., 2021). Real-ear measurements play a crucial role in verifying whether the hearing aid output aligns with the prescribed target, aiding in precise amplification adjustments (Denys et al., 2019). A 2010 survey revealed that clinicians conduct REMs mainly to ensure audibility of the speech spectrum (Mueller & Picou, 2010).

Despite the potential benefits, the integration of REM in clinical practice remains inconsistent. Studies reveal a lack of routine use of REMs by audiologists, indicating a gap between recommended practices and actual implementation (Christensen & Groth, 2008; Mueller, 2005). In a study conducted by Mueller (2005), it was revealed that among four groups of practising audiologists, only 38% – 42% of participants reported routinely conducting REMs, while a mere 21% of the final group incorporated REMs as part of their routine (Mueller, 2005). In the United States of America, Anderson et al. (2018) conducted an online survey that noted most participants in the study expressed a willingness to utilise REMs for fitting frequency-specific gain (Anderson et al., 2018). Similarly, a study conducted in 2010 revealed that most participants use verification procedures ‘sometimes’ (Mueller & Picou, 2010). Furthermore, in a low-income country, India, Easwar et al. (2013) found that only 25% of audiologists use some form of verification measures inconsistently (Easwar et al., 2013). The lack of verification has been attributed to factors such as equipment availability, cost, time constraints and clinician expertise (Easwar et al., 2013). Even within regions where REM is available, a significant proportion of professionals refrain from using the technique because of barriers such as limitations in time, a lack of confidence and gaps in knowledge (Amri et al., 2019; Mueller & Picou, 2010).

Two independent studies on resources available in the private and public sectors were conducted in South Africa in 2013 and 2022 (Bhamjee et al., 2022; Teixeira & Joubert, 2014). Findings specific to REM indicated that the primary reason for not conducting them was because of limited access to and a lack of verification equipment. A recent systematic review conducted by Almufarrij et al. (2021) emphasised the need for further investigation to comprehensively assess the efficacy of REM in aligning hearing aid amplification with validated prescription targets. This study focussed on the South African context and aimed to investigate the practices and perceptions of audiologists in both private and public sectors regarding hearing aid verification.

## Research methods and design

The study included a cross-sectional survey design utilising a mixed-method approach, containing both qualitative and quantitative aspects. The survey included four sections. Section A contained five questions on the demographic information of participants, section B of the survey comprised of four 5-point Likert scale questions and section C focused on verification practices and equipment. The survey concluded with Section D, which included two open-ended questions regarding participants’ opinions and perceptions of the barriers and facilitators for hearing aid verification in the South African context.

### Participants

Participants had to be audiologists registered with the Health Professions Council of South Africa (HPCSA) and practising in South Africa.

### Data collection

A survey was developed using the Qualtrics platform (Supplementary digital content A) and distributed across various social media platforms, alumni databases and organisations. The survey was accessible through a URL link and QR code available on an advert (Supplementary digital content B). Once the participants accessed the link, they were first required to provide informed consent. Participants were then asked whether they were practising in South Africa and registered with the HPCSA. If participants answered ‘yes’, they proceeded with the survey, and if participants answered ‘no’, the survey ended.

Participants who met the inclusion criteria completed section A which contained five questions on the demographic information of participants, such as gender and work setting (public hospital, private hospital, academic clinics, private practice or other). This was followed by section B of the survey which comprised of four 5-point Likert scale questions, ranging from 1 which indicated strongly agree and 5 indicating strongly disagree, of which participants rated their agreement to a series of statements regarding hearing aid verification, which was adapted from Amri et al. (2019). For Section C, the first question required participants to indicate how often they conduct hearing aid verification; if participants selected ‘not often’ or ‘never’, a follow-up question with a drop-down menu allowed the participants to select one or more reasons to substantiate their previous response. Thereafter, participants were directed to the final section of the survey. If participants selected ‘always’, ‘often’, or ‘sometimes’, eight follow-up questions were asked regarding: (1) access to verification equipment, (2) the state of verification equipment, (3) which client population they conduct verification on, (4) the participant’s feelings towards verification, (5) in which scenarios they conduct verification, (6) what participants felt would make them more confident when conducting verification, (7) the procedures they use to conduct verification and (8) their feelings towards verification. Participants could select more than one response for four out of the eight follow-up questions. The survey was concluded with section D where participants were required to answer two open-ended questions regarding their opinions on the barriers and facilitators to hearing aid verification in South Africa.

### Data analysis

Statistical Package for the Social Sciences (SPSS version 28) was used to descriptively analyse the quantitative data using frequency tabulations showing the number of participants (*n*) that selected a response, and percentages (%). Inductive thematic analysis, as described by Braun and Clarke (2006), was conducted on the survey’s open-ended questions. The thematic analysis occurred in the following steps. By thoroughly scanning through participant responses on an Excel spreadsheet, one researcher (D.J.) independently familiarised themselves with the responses obtained for the *facilitators* of hearing aid verification in South Africa. The other two researchers (J.M. & Z.B.) independently familiarised themselves with the responses obtained for *barriers* to hearing aid verification in South Africa. Coding for the data then occurred, with these three researchers coding independently. Extracts were used to form specific codes. Themes were then identified, where similar codes were grouped. A theme name was then decided upon. The researcher leading the *facilitators* section shared their findings with other researchers, reviewing and streamlining them to a few themes with extracted examples from the datasheet. The other two researchers leading the *barriers* section followed the same process.

### Ethical considerations

Ethical clearance to conduct this study was obtained from the University of Pretoria, Department of Speech-Language Pathology and Audiology Research Ethics Committee (reference no.: SLPA2023/01).

## Results

### Participant characteristics

Seventy-eight audiologists, of which 94.9% were female (*n* = 74) participated in the study (Table 1). Years of experience in the field ranged from less than a year up to 34 years. More than half (51.3%; *n* = 40) of the audiologists reported that they were based in the Gauteng province, followed by the Western Cape (15.4%; *n* = 12), with no responses from the Free State. Several participants reported practising across multiple settings (school, clinic, etc.); the majority (69.2%) were based in private practice.

**TABLE 1:** Demographic information of participants (*N* = 78).

Demographic information	%	*n*
**Gender**		
Male	3.8	3
Female	94.9	74
Prefer not to say	1.3	1
**Years of experience**		
< 1	5.2	4
1–5	25.6	20
6–10	15.4	12
11–15	19.2	15
16–20	15.4	12
> 20	19.2	15
**Province**		
Eastern Cape	5.1	4
Free State	0.0	0
Gauteng	51.3	40
KwaZulu-Natal	10.3	8
Limpopo	5.1	4
Mpumalanga	6.4	5
Northern Cape	2.6	2
North West	3.8	3
Western Cape	15.4	12
**Area**		
Rural	1.3	1
Urban	79.5	62
Semi-urban	19.2	15
Employment setting*		
Public hospital or clinic	16.7	13
Private hospital	23.1	18
Private practice	69.2	54
Academic	15.4	12
Corporate or hearing aid manufacturer	5.1	4
Retail	2.6	2
Schools for the deaf or LSEN	3.8	3

*Source:* Moll, J., Burger, Z., Jacobs, D.M.P., & Mothibe, R.P. (2023). *Hearing aid verification: practices and perceptions of South African audiologists*. Dissertation. University of Pretoria

* , More than one option could be selected.

### Perceptions of hearing aid verification

**FIGURE 1:**
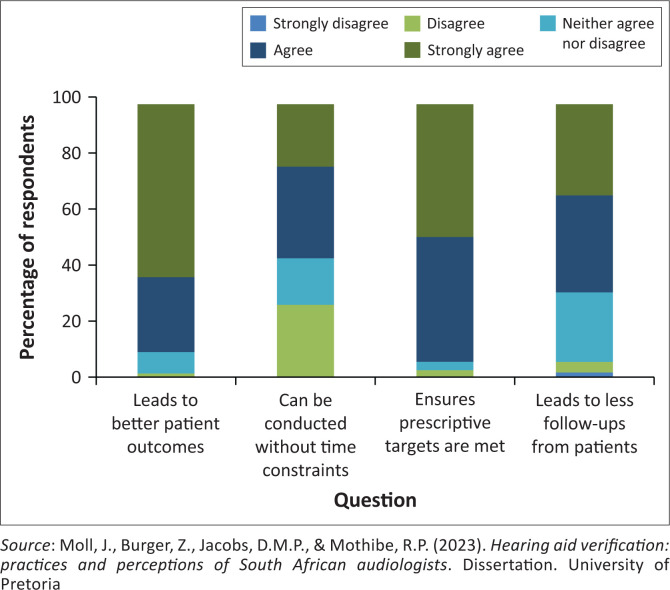
Participant perceptions on the use of hearing aid verification (*N* = 76).

Most of the participants strongly agreed or agreed that hearing aid verification was beneficial. Only one participant strongly disagreed, when asked whether hearing aid verification results in fewer follow-up appointments from patients (Figure 1).

### Hearing aid verification practices

Of the total, 81.0% of audiologists reported performing hearing aid verification on all clients, including both adults and children. In contrast, 10.3% of audiologists exclusively conduct verification on adults, while 6.9% of audiologists solely focus on children under the age of 12.

Less than half (47.4%) of audiologists indicated that they always conduct hearing aid verification on all clients, 19.7% indicated they do verification often and 9.2% do it sometimes. More than three in every four audiologists (86.6%) who conduct verification, indicated that they do so during every client’s first fit, while half (53.8%) of participants conduct verification when a problem arises (Table 2).

**TABLE 2:** Participants’ reasons for conducting or not conducting verification.

Statement	% of responses	*n*
Reasons for not conducting verification (*n* = 19*)		
Improper equipment	40.7	11
It is not a standard practice to verify hearing aids in my work setting	25.9	7
It is not crucial as I can use other measures to optimise hearing aid fitting	18.5	5
No access to verification equipment	11.1	2
I do not have enough time to do it	3.7	1
I work in corporate and conduct verification when the client requests support. I do not work directly with the patients	3.7	1
Reasons for conducting verification (*n* = 52*)		
Every client’s first fit	86.6	45
Whenever a problem arises	53.8	28
When acoustic parameters change or adjustments are made	17.3	9
Follow-up appointments	9.6	5
Annual hearing test	3.8	2
After hearing aids have been serviced	1.9	1
Flight medicals	1.9	1

*Source:* Moll, J., Burger, Z., Jacobs, D.M.P., & Mothibe, R.P. (2023). *Hearing aid verification: practices and perceptions of South African audiologists*. Dissertation. University of Pretoria

* , Multiple answers could be selected; alternatively, participants could choose ‘other’ and provide a reason.

Of the total, 23.0% indicated they seldom or never use verification, a follow-up question to these participants (Table 2) indicated that nearly half (40.7%) are unable to verify because of improper equipment while 11.1% indicated no access to verification equipment.

A wide variety of equipment was used for verification (Table 3). Nearly all (90.0%) of the participants who had access to equipment (*n* = 50) reported that the equipment was functional and reliable. The remaining 6.0% of participants (*n* = 3) indicated functional equipment but were unable to do Real-Ear-to-Coupler Difference (RECD), whereas 4.0% indicated that their equipment is not functioning or reliable (*n* = 2).

**TABLE 3:** What verification equipment is used (*N* = 50*).

Verification equipment	% of responses	*n*
**Audioscan**	42	21
(1) *Axion*	*8*	*4*
(2) *Verifit*	*22*	*11*
(3) *Axion and verifit*	*12*	*6*
Otometrics Aurical Freefit	18	9
Interacoustics Callisto	6	3
Fonix	2	1
Siemens Unity	16	8
MedRX REM	4	2
Otosuite verification	2	1
Audidata Primus	6	3
Unspecified REM	8	4
Unspecified Speech Mapping	6	3

*Source:* Moll, J., Burger, Z., Jacobs, D.M.P., & Mothibe, R.P. (2023). *Hearing aid verification: practices and perceptions of South African audiologists*. Dissertation. University of Pretoria

Note: Italic values and names represent the certain verification equipment.

REM, real-ear measurements.

* , Participants were prompted to list the equipment available to them with the option to provide multiple answers.

**FIGURE 2:**
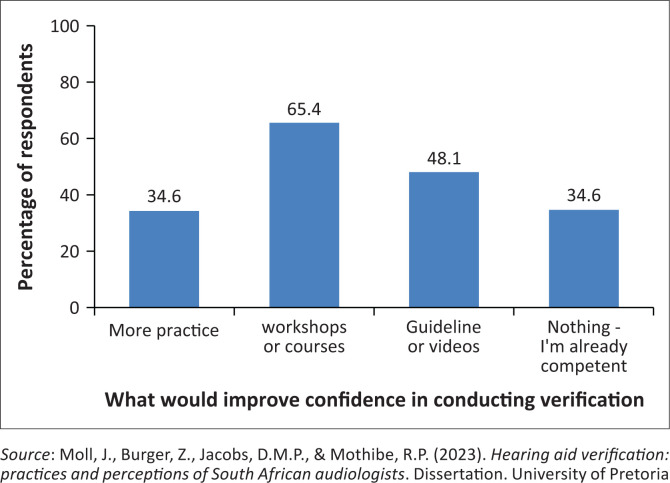
Participant responses on what would make them feel more confident in conducting hearing aid verification (*N* = 52). Participants were allowed to select multiple answers from four available options.

Most participants who indicated they do verification (92.3%) were confident in their skills to conduct verification using REM. However, only 46.2% of participants were confident in their skills to conduct RECD in situations where REMs were not feasible. Of the total, 19.2% of participants indicated that the training during their education was sufficient to conduct verification while only 11.5% of participants experienced that the training they received during their community service was sufficient for them to conduct hearing aid verification. A total of 65.4% of audiologists indicated that attending workshops or courses would make them feel more confident in conducting hearing aid verification. However, 34.6% of audiologists indicated that they are already competent in their ability to perform hearing aid verification and do not require further training (Figure 2).

Table 4 summarises the various procedures used by audiologists for hearing aid verification in South Africa. A total of 88.5% of participants indicated that they use different stimulus levels to measure the hearing aid gain while REM is utilised by 80.8% of audiologists and RECD is utilised by 32.7% of audiologists.

**TABLE 4:** Participant responses on hearing aid verification practices in South Africa (*N* = 52).

Procedure used during hearing aid verification	% of responses	*n* *
I use different stimulus levels to measure the hearing aid gain	88.5	46
Real-ear measurements (REM)	80.8	42
I consider speech mapping a valid aspect of my approach to hearing aid verification	78.8	41
I measure the maximum power output (MPO) level provided by hearing aids	65.4	34
Real-ear to coupler difference (RECD)	32.7	17
Verification of frequency compression	5.8	3
Free field speech and speech in noise testing	1.9	1
Occlusion effect	1.9	1

*Source*: Moll, J., Burger, Z., Jacobs, D.M.P., & Mothibe, R.P. (2023). *Hearing aid verification: practices and perceptions of South African audiologists*. Dissertation. University of Pretoria

* , Multiple answers could be selected; alternatively, participants could choose ‘other’ and provide a reason.

### Thematic analysis of hearing aid verification facilitators and barriers

Qualitative analysis revealed 10 themes relating to facilitators towards hearing verification and five themes related to barriers, as indicated in Table 5.

**TABLE 5:** Qualitative themes identified for the benefits, facilitators and barriers to conducting hearing aid verification in the South African context.

Themes	Examples
**Facilitators**	
Counselling tool	‘Our REMs system can also simulate what a patient hears which is a good counselling tool for family members.’ (Participant 73, Female, Private practice)
Especially with children	‘I believe verification is crucial for kids who cannot give proper feedback. And therefore, beneficial when fitting kids. With adults, it’s not necessary.’ (Participant 38, Female, Private practice)
Objective validation	‘To provide objective, reliable, and accurate verification of what the hearing aid is really doing in the patient’s ear.’ (Participant 19, Female, Private practice)
When there is a language barrier	‘In the South African context, we have language barriers, prohibiting effective feedback from the patient regarding loudness.’ (Participant 8, Male, Private practice)
Reduced follow-up appointments	‘Verification of hearing aid programming helps to eliminate frequent visits to the audiologist for adjustment of hearing aid settings.’ (Participant 40, Female, Public and academic)
Improved patient outcomes	‘The more accurate we set the hearing aid during the first fit, the more satisfied the patient is with their hearing aid.’ (Participant 40, Female, Public and academic)
Best practice	‘Verification forms an integral part of an evidence-based hearing aid fitting.’ (Participant 50, Female, Private practice)
Accounts for individual ear differences	‘It takes each person’s unique ear canal characteristics into account when adjusting hearing aid gain and output.’ (Participant 19, Female, Private practice)
Gives clinician confidence	‘It provides me with confidence that I am fitting my patient to the best of my abilities and does not feel that I’m shooting in the dark.’ (Participant 12, Female, Academic)
Difficult-to-test population cases	‘Better prescriptive targets for complex patients.’ (Participant 14, Female, Private practice)
**Barriers**	
Cost of and access to verification equipment	‘The majority of private practices and public Audiology departments do not have verification equipment due to the cost of the equipment.’ (Participant 39, Female, Private practice)
Insufficient skills	‘Lack of knowledge on the benefit of verification. The other barrier is the knowledge of how to conduct verification. In my opinion, we did not receive enough training or opportunities to perform verification at my Academic Institution. What I learned, I learned at first employer’s private practice more than what I learned at my Academic Institution or in Community service.’ (Participant 12, Female, Academic)
Time constraints	‘Can be timely, especially when there is a language barrier as the whole fitting takes longer.’ (Participant 41, Female, Private practice)
Interrupted electricity supply	‘Loadshedding and the costs.’ ‘Uninterrupted electricity supply.’ (Participant 35, Female, Private practice)
Trust in the first fit of the hearing aid manufacturer	‘Trust that the hearing aid manufacturer’s software will meet prescriptive target settings.’ (Participant 17, Female, Other)

*Source:* Moll, J., Burger, Z., Jacobs, D.M.P., & Mothibe, R.P. (2023). *Hearing aid verification: practices and perceptions of South African audiologists*. Dissertation. University of Pretoria

REM, real-ear measurements.

## Discussion

According to ASHA’s Scope of Practice in Audiology document, clinicians should employ evidence-based techniques in practice that are supported by research. Real-ear measurements is considered to be such for hearing aid fittings (American Speech-Language-Hearing Association 2018, 2023). American Speech-Language-Hearing Association also stipulates that as part of a comprehensive audiological rehabilitation programme, the clinician should verify, among others, technological interventions (American Speech-Language-Hearing Association, 2018). Furthermore, the British Society of Audiology (2018) strongly recommends the use of REM measurements as the starting point for a hearing aid fitting (British Society of Audiology, 2018).

The practices and perceptions of South African audiologists regarding hearing aid verification were surveyed in this study. Overall, there was a strong consensus that conducting hearing aid verification leads to improved patient outcomes, guarantees that hearing aids reach prescriptive targets and reduces follow-ups from patients. These were later identified as themes for facilitators of verification, categorised as ‘Improved outcomes’ and ‘Best Practice’. The study revealed that one in four (25.6%) audiologists agreed that verification contributes to time constraints, which was identified as a barrier in the final open-ended question ‘Time constraints’. This echoes the findings by Folkeard et al. (2018), which describe the impact of hearing aid verification time requirements on hearing aid appointments and clinician perceptions. In the study conducted by Amri et al. (2019), 37% of participants indicated a lack of time as a reason for not conducting verification, while only 3.7% of the participants in this study shared that view. Thus, the time required to perform verification is an important clinical consideration. This, along with the cost-benefit of conducting verification, should be investigated as highlighted by Almufarrij et al. (2021).

Numerous participants indicated that they never perform hearing aid verification or perform it rarely. When prompted to give reasoning behind not conducting hearing aid verification, the majority opted for ‘improper equipment’. Hence, it appears that many of the participants who do not conduct hearing aid verification do so because of improper equipment or lack thereof. This was established as a theme for barriers to conducting hearing aid verification. Within this study, 51.8% of participants indicated issues of improper equipment or lack of access to verification equipment, with the majority of participants working in private practice. The study conducted by Bhamjee et al. (2022) revealed that 75.6% of audiologists employed in the public sector of South Africa also lack access to adequate verification equipment. Therefore, the issue of verification equipment and the expense thereof is prevalent in both private and public healthcare sectors in South Africa.

Other studies have also pointed out the challenge of access to appropriate verification equipment. Amri et al. (2019) found a significant number of participants (34.2%) needed help with properly functioning verification equipment for real ear aided response (REAR) or RECD measurements. Their study highlighted that many participants faced challenges with uncooperative paediatric patients during verification, a factor this study did not fully explore as the focus was on verification procedures across all patient populations in South Africa, not specifically on paediatric cases.

Interestingly, participants in our study recognised the benefit of conducting RECD in challenging cases, as evident from the theme, ‘Difficult-to-test population cases’ identified as a facilitator. However, not all participants have access to verification equipment that can perform RECD, accounting for 10% of these participants. A similar percentage of participants in both studies (46.2% and 43.5%) reported a lack of confidence in conducting RECD (Amri et al., 2019). Various types of verification equipment are available on the market. This study indicated Audioscan as the most used verification equipment by the participants (42%).

A notable theme for the barriers to conducting hearing aid verification was the reliance of participants on hearing aid manufacturers’ software (first-fit) to meet the prescribed target settings. This theme correlates with the findings of Abrams et al. (2012), which indicate that with a first-fit method, it is generally expected that the prescribed responses will closely match with the underlying prescriptions used as there is no verification process to ensure that the hearing aids will meet the prescribed targets (Abrams et al., 2012). However, Abrams et al. (2012) revealed a significant difference between the first-fit approach and the verified measurements, as the first-fit had a greater deviation from the target (Abrams et al., 2012).

In this study, 19.2% of participants expressed that the education they received was sufficient to conduct verification. In contrast, Amri et al. (2019) found that 34.3% of participants expressed that institutional training was sufficient for them to perform verification. Furthermore, 34.6% of participants in this study indicated that they feel competent and do not require additional training to improve their verification skills. On the contrary, only one participant in the study of Amri et al. (2019) indicated that they are already confident in their verification methods, and do not need further training. This data indicated that a higher confidence level exists among South African audiologists. Differences in training curriculums or guidelines possibly account for the discrepancy observed between the two studies, as most participants in the study by Amri et al. indicated the need for further training (Amri et al., 2019). The study revealed that 65.4% of South African audiologists with access to verification equipment feel the need for additional training or exposure to gain confidence in verification procedures. These findings highlight the necessity for additional and/or improved training opportunities for audiologists in the practice of hearing aid verification.

Qualitative themes identified as facilitators include increased clinician confidence during hearing aid fitting, along with the perception that hearing aid verification is objective and evidence-based. In contrast, reported barriers to conducting verification included the impact of interrupted electricity supply because of ‘loadshedding’ in South Africa. Public health facilities highlighted time constraints and a lack of equipment as primary barriers, with some mentioning the required headcount leaving limited time per appointment.

This study presented with some limitations. First of all, the sample size was limited to a total of 78 participants with only 66 completing all sections of the survey because of options ‘never’ or ‘seldom’ resulting in section C being skipped. Additionally, the distribution of respondents was also limited in terms of sex distribution, with only three males participating; however, this is representative of the field of audiologists in South Africa, which is predominantly female (Pillay et al., 2020). The study shows a possibly skewed sample, likely because of the study invitation attracting mainly SA audiologists who perform REMs, while potentially discouraging those who do not perform REMs from participating.

## Conclusion

This study underscores the value audiologists in South Africa place on hearing aid verification by investigating their verification practices in different settings. Clinicians are fully aware of the importance of hearing aid verification, appreciating its potential to improve patient outcomes, boost clinician confidence, reduce follow-up visits and bridge language barriers. They recognise its value as a counselling tool, adapting care to difficult-to-test populations. Audiologists primarily use different stimulus levels, REM, speech mapping, maximum power output (MPO) and RECD as verification methods. Nevertheless, frequency compression, free field speech, speech in noise and assessing the occlusion effect are procedures less frequently used for verification. Nonetheless, barriers such as time constraints, equipment accessibility, cost and interrupted electricity supply, among others, prevent clinicians from conducting hearing aid verification. The evident gap in educational training emphasises the potential of supplemental courses to enhance proficiency. Collaborative initiatives are essential to address the noted barriers and promote optimal hearing aid fitting practices, elevating patient experiences and overall hearing healthcare standards.
